# Paediatric Wolfram syndrome Type 1: should gonadal dysfunction be part of the diagnostic criteria?

**DOI:** 10.3389/fendo.2023.1155644

**Published:** 2023-06-13

**Authors:** Giulio Frontino, Raffaella Di Tonno, Marianna Rita Stancampiano, Francesca Arrigoni, Andrea Rigamonti, Elisa Morotti, Daniele Canarutto, Riccardo Bonfanti, Gianni Russo, Graziano Barera, Lorenzo Piemonti

**Affiliations:** ^1^ Department of Pediatrics, Scientific Institute for Research, Hospitalization and Healthcare (IRCCS) San Raffaele Hospital, Milan, Italy; ^2^ Diabetes Research Institute, Scientific Institute for Research, Hospitalization and Healthcare (IRCCS) San Raffaele Hospital, Milan, Italy; ^3^ Faculty of Medicine and Surgery, Vita-Salute San Raffaele University, Milan, Italy; ^4^ San Raffaele Telethon Institute for Gene Therapy, Scientific Institute for Research, Hospitalization and Healthcare (IRCCS) San Raffaele Hospital, Milan, Italy; ^5^ Pediatric Immunohematology Unit and BMT Program, Scientific Institute for Research, Hospitalization and Healthcare (IRCCS) San Raffaele Hospital, Milan, Italy

**Keywords:** Wolfram syndrome, Wolfram syndrome 1 (WFS1), monogenic diabetes, gonadal dysfunction, hypogonadism, hypergonadotropic hypogonadism

## Abstract

**Aims:**

Wolfram Syndrome Spectrum Disorder (WFS1-SD), in its “classic” form, is a rare autosomal recessive disease with poor prognosis and wide phenotypic spectrum. Insulin dependent diabetes mellitus (DM), optic atrophy (OA) diabetes insipidus (DI) and sensorineural deafness (D) are the main features of WFS1-SD. Gonadal dysfunction (GD) has been described mainly in adults with variable prevalence and referred to as a minor clinical feature. This is the first case series investigating gonadal function in a small cohort of paediatric patients affected by WFS1-SD.

**Methods:**

Gonadal function was investigated in eight patients (3 male and 5 female) between 3 and 16 years of age. Seven patients have been diagnosed with classic WFS1-SD and one with non-classic WFS1-SD. Gonadotropin and sex hormone levels were monitored, as well as markers of gonadal reserve (inhibin-B and anti-Mullerian hormone). Pubertal progression was assessed according to Tanner staging.

**Results:**

Primary hypogonadism was diagnosed in 50% of patients (n=4), more specifically 67% (n=2) of males and 40% of females (n=2). Pubertal delay was observed in one female patient. These data confirm that gonadal dysfunction may be a frequent and underdiagnosed clinical feature in WFS1-SD.

**Conclusions:**

GD may represent a frequent and earlier than previously described feature in WFS1-SD with repercussions on morbidity and quality of life. Consequently, we suggest that GD should be included amongst clinical diagnostic criteria for WFS1-SD, as has already been proposed for urinary dysfunction. Considering the heterogeneous and elusive presentation of WFS1-SD, this clinical feature may assist in an earlier diagnosis and timely follow-up and care of treatable associated diseases (i.e. insulin and sex hormone replacement) in these young patients.

## 1 Introduction

Classic Wolfram Syndrome Spectrum Disorder (WFS1-SD) is a rare genetic condition with autosomal recessive transmission and an estimated prevalence of 1:160.000 - 1:770.000 (1, 2). WFS1-SD is caused by mutations in the gene encoding wolframin (WFS1), a protein primarily located in the endoplasmic reticulum (ER) (2). Consequent dysregulation in ER and cytoplasmic Ca2+ homeostasis seems to be the main process leading to apoptosis and degeneration involving β- and neural cells (3–7). Main clinical features of classic WFS1-SD include diabetes mellitus (DM) optic atrophy (OA) diabetes insipidus (DI), and sensorineural deafness (D), hence the acronym DIDMOAD (2, 8). Nonetheless, clinical presentation is extremely variable and often complicated by neurological, urological, psychiatric and other endocrine dysfunctions (2, 4, 8–10). To date, mutations of WFS-1 with dominant transmission have been described (11). Classified as non-classic WFS1-SD, these defects determine deafness (sometimes associated with milder optic atrophy), diabetes alone or congenital cataracts (5, 12, 13).

Currently, there is no treatment able to delay, stop, or reverse the natural history of disease (5). Novel and already marketed/repurposed drugs are being assessed as possible disease modifying agents (2, 5, 7).

Gonadal dysfunction, mainly hypogonadotropic hypogonadism, has been reported and is to date considered a minor clinical feature in WFS1-SD patients (14, 15). Hypergonadotropic hypogonadism has also been recently described (5, 14–18).The prevalence of both primary and secondary hypogonadism in WFS1-SD varies widely from different reports, ranging from 7.1% to almost 30% (16, 19, 20).

To date, gonadal failure has been observed more frequently in young male adults with heterogeneous manifestations. In males, associated features described with hypogonadism included atrophic testes, small penis, erectile dysfunction, gynecomastia and poor secondary sexual characteristics (2, 5, 15, 17, 19, 21). Females may often generally present with primary amenorrhoea (22). Interestingly, the first case of primary hypergonadotropic hypogonadism in a female patient with confirmed WFS1-SD has been recently described in a 16 year adolescent (23). Pubertal delay is often observed in WFS1-SD adolescents alongside growth delay due to hypopituitarism (15, 23). Nevertheless, cases of successful pregnancy and delivery exist and first reports of male fertility date back to a decade ago (15, 24, 25).

The aim of our study was to assess gonadal function in a small cohort of eight paediatric patients (3 male and 7 female) with WFS1-SD undergoing regular follow-up at IRCCS San Raffaele Hospital in Milan.

## 2 Materials and methods

### 2.1 Population

Our study group includes eight patients between 3 and 16 years old. Seven patients have been diagnosed with genetically confirmed classic WFS1-SD and one (the youngest female) non-classic WFS1-SD. All patients are currently undergoing an off-label treatment with subcutaneous liraglutide alongside classic treatments required for diseases associated with the syndrome (i.e. insulin therapy, growth hormone replacement therapy). Patients 1 to 4 were previously included in a published case series assessing safety and tolerability of liraglutide in paediatric WS (26)

### 2.2 Diagnostic assessment

Hypothalamic-pituitary-gonadal axis was assessed by serum gonadotropin (luteinizing hormone, LH and follicle-stimulating hormone, FSH) and sex hormones (testosterone in males and oestrogen in females) 1evels. Serum anti-Mullerian hormone (AMH) and inhibin-B (INHBB) were assessed in male and female patients respectively to investigate Sertoli cell function and ovarian reserve (27–30). LH, FSH, testosterone, oestradiol and AMH in patients serum were measured by electrochemiluminescence (ECLIA) using an automated analyzer (Roche COBAS^®^). Inhibin-B serum concentration was assessed by automated enzyme-linked immunosorbent assay (DYNEX DSX™ Automated ELISA).

Pubertal development was assessed according to Tanner staging (A, axillary hair; PH, pubic hair; G, male external genitalia; B, female breast) (31).

## 3 Results

### 3.1 Case description

Patient 1 (male) presented with hyperopia and astigmatism at age 6. Shortly after, he developed bilateral neurosensorial hearing loss requiring use of hearing aids. He was diagnosed with non-autoimmune type 1 diabetes (naT1D) at age 6.7 years old and started treatment with multiple daily insulin injections (MDI). WFS1-SD diagnosis was confirmed by genetic analysis showing a double heterozygous WFS1 gene variant (c.409_424dup16; p.Val142fs∗251 and c.1628T>G; p.Leu543Arg). At the age of 8.3 years, diabetes insipidus was diagnosed and treatment with desmopressin was started. Magnetic resonance imaging (MRI) showed OA and mild brainstem hypoplasia.

Patient 2 (female) had an unremarkable medical history except for transient mild speech delay. T1D was diagnosed at age 5.2 years. At age 8.7 years, genetic analysis identified two previously unreported likely pathogenic variants (c.316-1G>A; c.757A>T and p.Lys253Ter; splice site disruption and premature stop codon respectively). MRI showed slight atrophy of brainstem and optic nerves.

Patient 3 (male) had an unremarkable past medical history except for a transient mild motor delay. T1D was diagnosed at 9 years. Genetic confirmation of WFS1-SD was established by NGS (Next Generation Sequencing) showing a double heterozygous missense variant (c.605A>G; p.Glu202Gly and c.1289C>T; p.Ser430Leu). Optical coherence tomography (OCT) scans highlighted OA at 10.7 years of age, later confirmed by MRI.

Patient 4 (male) had a prior unremarkable medical history and was diagnosed with T1D at age 12.3 yrs. Aged 13.4, OA was confirmed at OCT scan. Gene sequencing revealed heterozygous WFS1 variants resulting in a premature stop codon and missense mutation (c.387G>A; p.Trp129 and c.1675G>C; p.Ala559Pr respectively). Reduce volume of brainstem at MRI was found.

Patient 5 (female, sister of patient 3) underwent NGS analysis at age of 10.8 years after her brother’s diagnosis and the same WFS1 gene variants were confirmed. Interestingly, sequencing also reported missense heterozygous variant (c.778G>C; p.Glu260Gln) of paternal inheritance in KCNQ4 gene, associated with neurosensorial hearing loss. Her past medical history included hyperopia and strabismus requiring corrective lenses, diagnosis of bilateral congenital hypoacusia at 7.3 years of age requiring hearing aids and mild speech delay for which she underwent speech therapy. Bilateral optic nerve, pons and cerebellar atrophy were found on MRI assessment at age 10.8. At age of 11.7 years glucose intolerance was observed at mixed meal tolerance test (HbA1c 31 mmol/mol; 5%).

Patient 6 (female) presented with progressive neuromotor delay since birth and bilateral congenital cataract treated with surgery at 1.1 years. Shortly after naT1D was diagnosed, NGS analysis showed a *de novo* heterozygous WFS1 gene mutation (c2425G>A, p.Glu809Lys), previously described as pathogenetic in autosomal dominant non-classic WFS1-SD. At 3.1 years severe neurosensorial hearing loss was diagnosed and hearing aids were prescribed. Patient 6 was then referred to our centre, where an advanced hybrid closed loop system was started. Growth hormone deficit was also diagnosed and replacement therapy was started. Brain MRI showed a normal pituitary gland.

Patient 7 (female) had an unremarkable past medical history until 5.5 years of age when she presented with progressive visual impairment. OA was found at OCT assessment and genetic analysis for Leber optic atrophy was negative. At the age of 8.7 years classic WFS1-SD was diagnosed (two heterozygous variants, c.108delG in exon 2 and c.2206G>A in exon 8). At the same timepoint she presented fasting hyperglycaemia (300 mg/dl) and increased haemoglobin A1c (9.8%). The patient was transferred to our Centre for follow-up and treatment

Patient 8 (female) was born from consanguineous parents. T1D was diagnosed at 4.2 years in her country of origin (Egypt). Afterwards, her medical history was complicated by bilateral cataracts (5.5 years of age) for which she underwent surgical correction. Genetic analysis performed at 9 years of age showed the presence of a single likely pathogenic homozygous variant in WFS1 (c.2140G>C, p.Asn714Asp) confirming classic WFS1-SD. Screening for associated diseases showed mild sensorineural hearing loss. The girl was referred to our centre for periodic follow-up

**Table 1** summarizes patient genotype and WFS1-SD related phenotype.

**Table 1:** General pathognomonic characteristics of WFS and genetic mutations of all patients.

	Sex	Origin	DM Diagnosis (age)	WFS Diagnosis (age)	Genetic	Clinical characteristics	Start liraglutide (age)
**Patient 1**	M	Italian	6 y 8 m	8 y	Double heterozygosity: c.409_424dup16; p.Val142fsX251 and c.1628T>G; p.Leu543Arg	DM OA DI HD	11 y 3 m
**Patient 2**	F	Italian	5 y 2 m	8 y 7 m	Double heterozygosity: c.316-1G>A; c.757A>T. p.L	DM OA	10 y 7 m
**Patient 3**	M	Italian	9 y	10 y 7 m	Double heterozygosity: c.605A>G p.G1u 202G1y e - c.1289C>T p.Ser430leu.	DM OA	12 y 3 m
**Patient 4**	M	Italian	12 y 3 m	13 y 4 m	Double heterozygosity: c387G>A p.Trp129X e - c.1675G>C p.Ala559Pr.	DM OA	14 y
**Patient 5**	F	Italian		10 y 8 m	Double heterozygosity: c.605A>G p.G1u 202G1y e - c.1289C>T p.Ser430leu.	GI OA HD	11 y 3 ms
**Patient 6**	F	Albanian	1 y 6 m	1 y 6 m	Dominant: c2425 G>A, p.Glu809Lys	DM CC HD GHD	3 y 3 m
**Patient 7**	F	Hispanic	8 y 7 m	8 y 7 m	Double heterozygosity c.108delG in exon 2 and c.2206G>A	DM OA	8 y 7 m
**Patient 8**	F	Egyptian	4 y	9 y	Single homozygous variant c.2140G>C, p.Asn714Asp	DM CC HD	9 y 9 m

DM, diabetes mellitus; OA, optic atrophy; DI, diabetes insipidus; HD, hearing defects; UD, renal or urological problems; GI, glucose intolerance; GHD, growth hormone deficit; CC, congenital cataract.

### 3.2 Gonadal assessment

Patient 1 showed an increased value of LH and FSH first at 12 years and 8 months of age, confirmed at yearly clinical follow-up (see **Table 2**). Clinical examination showed a regular progression of pubic hair and genital virilisation (according to Tanner staging). However, testicular volumes remained lower than expected for age and pubertal staging (4 cc bilaterally at last evaluation; 15 yo) and inhibin-B still remained unmeasurable. Hormonal exams were diagnostic for hypergonadotropic hypogonadism, and testosterone levels are being assessed on regular follow-up to evaluate the need for testosterone replacement therapy (see **Table 2**).

**Table 2:** Gonadal and endocrinological assessment of all patients.

	Sex	Age	LH (mU/ml)	FHS (mU/ml)	E	T (ng/ml)	AMH (mcg/L)	Inhibin B (pg/ml)	Tanner stage	Hight (SDS)	Weight (SDS)	BMI (SDS)
**Patient 1**	M	12 y 8 m 13 y 6 m 14 y 3 m 15 y	65,6 29,2 18,9 23,3	16,6 55,7 58 54,1		2 3,11 3,27 2,18	3,96 7,21 3,66 2,9	<7 <7 <7 <7	A2; P2-3; G2 A3; P3; G3 A3; P4; G4 A3; P5; G4	1.01 0.56 0.13 0.25	0.83 0.13 -0.52 -0.69	0.48 -0.30 -0.88 -0.78
**Patient 2**	F	10 y 8 m 11 y 11 y 4 m 12 y 4 m 13 y 8 m	<0.3 <0.3 <0.3 <0.3 1.1	2.7 1.8 3.1 2.8 5.1	< 5 14		0.83 0.57 0.22		A1; B1; P1 A1; B1; P1 A1; B1; P1 A1; B1; P1-2 A2; B2; P3	-0.09 -0.06 -0.31 -0.54 0.10	-1.39 -1.76 -1.77 -2.16 -1.95	-2.07 -2.76 -2.55 -2.87 -2.69
**Patient 3**	M	12 y 4 m 13 y 1 m 13 y 8 m 14 y 6 m	1,5 4,5 6.3 4.7	1.8 2.1 3 3.3		0,38 1,69 2,93 3,63	22.5 7.77	161,9	A2; P2; G2 A2; P2; G2 A3; P3; G3 A3; P3; G3	-0.92 0.90 -0.99 -1.13	0.87 0.77 1.15 1.03	1.59 1.47 1.92 1.83
**Patient 4**	M	14 y 14 y 7 m 15 y 3 m 16	13.1 12.9 10.3 19.8	33.7 32.9 29.2 33.6		5.54 4.77 3.78 6.51	12.7 11.1 8.29	<7 <7 <7	A2; P2; G3 A3; P3; G4 A3; P4; G5 A3; P4; G5	1.02 0.71 0.57 0.56	0.05 -0.50 -0.72 -0.45	-0.74 -1.12 -1.37 -0.81
**Patient 5**	F	10 y 10 m 11 y 8 m 12 y 1 m	9.1 7 11.3	6.2 10.1 9	43 56		1.55 1.25		A2; B2-3; P2 A2; B3; P3 A3; B3; P3-4	0.94 0.73 0.69	0.04 -0.34 -0.80	-0.53 -0.97 -1.60
**Patient 6**	F	3 y 3 m 3 y 9 m	5.2 3.3	44.5 35.3	<5 <5		<0,01 <0,01		A1; B1; P1 A1; B1; P1	-3.10 -3.10	-3.23 -4.30	-1.82 -2.06
**Patient 7**	F	8 y 8 m	<0.3	1.9	<5		3.62		A1; B1; P2	-0.78	-1.61	-1.79
**Patient 8**	F	9 y 11 m	24.6	37.4			0.01		A1; B1; P1	-0.73	-0.95	-0.83

LH, Luteinizing hormone; FSH, Follicle-Stimulating hormone; AMH, Anti- Mullerian hormone; BMI, Body Mass Index; SDS, Standard Deviation Score.

Patient 2 presented a pubertal delay, with first evidence of breast budding around 13 years of age. Periodical laboratory assessment showed prepubertal LH, FSH and oestradiol values compatible with Tanner staging until the last follow-up (13 years and 8 months), when pubertal progression was clinically evident (Tanner stage: A2, B2, PH3) with concomitant gonadotropin rising. However, at the same age, serum AMH values were still in the lower range, possibly suggesting a early-stage primary ovarian insufficiency (POI; see **Table 2**).

Patient 3 presented a normal progression of pubertal development, but a testicular asymmetry, with a right testis of 5 cc and left testis of 12 cc at 13 years and 8 months of age. The ultrasound of the right testis, performed at the same timepoint, showed initial signs of fibrosis of the seminiferous tubules. Gonadotropins, testosterone, inhibin-B and AMH values remained normal for age and pubertal stage duwholeg the all follow-up (12 y 4 m – 14 y 6 m, see **Table 2**).

Patient 4 showed an increased value of LH and FSH first at 14 years of age, confirmed at yearly clinical follow-up (see **Table 2**). Clinical examination showed a regular progression of pubic hair and genital virilisation (according to Tanner staging). However, testicular volumes remained lower than expected for age (4 cc) and pubertal staging and inhibin-B still remained unmeasurable at last evaluation (16 year old). Hormonal exams were diagnostic for hypergonadotropic hypogonadism, but testosterone levels always remained in the normal range for age and pubertal stage, with no currently need for hormonal replacement therapy (see **Table 2**)

Patient 5 showed normal progression of pubertal development with appropriate gonadotropin and AMH values for age and sex at all timepoints (10 y 10 m, 11 y 8 m and 12 y 1 m; see **Table 2**).

Patient 6 showed an increased value of LH and FSH since the first evaluation at 3 years and 3 months of age (see **Table 2**). Hormonal exams, performed at the same age and repeated 6 month later, were diagnostic for early hypergonadotropic hypogonadism and AMH values confirmed the diagnosis of primary ovarian insufficiency at a very early age (see **Table 2**).

Patient 7, assessed at 8 year and 8 months of age, was prepubertal. Serum gonadotropins and AMH values were normal for age and sex. (see **Table 2**).

Patient 8 showed an increased value of LH and FSH since the first evaluation at almost 10 years of age (see **Table 2**). Hormonal exams, performed at 9 years and 11 months of age, were diagnostic for early hypergonadotropic hypogonadism and AMH values confirmed the diagnosis of premature ovarian insufficiency (see **Table 2**).

**Figure 1:**
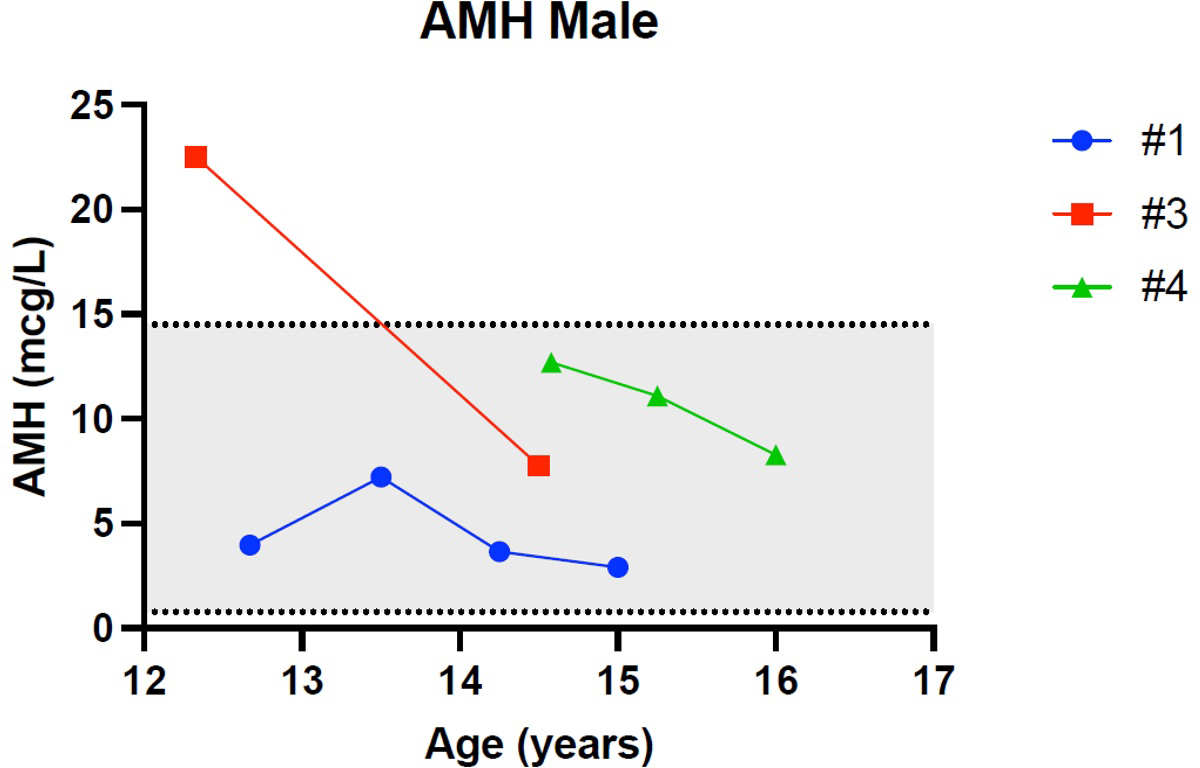
Trend of anti-Mullerian hormone in males. # stands for “patient”.

**Figure 2:**
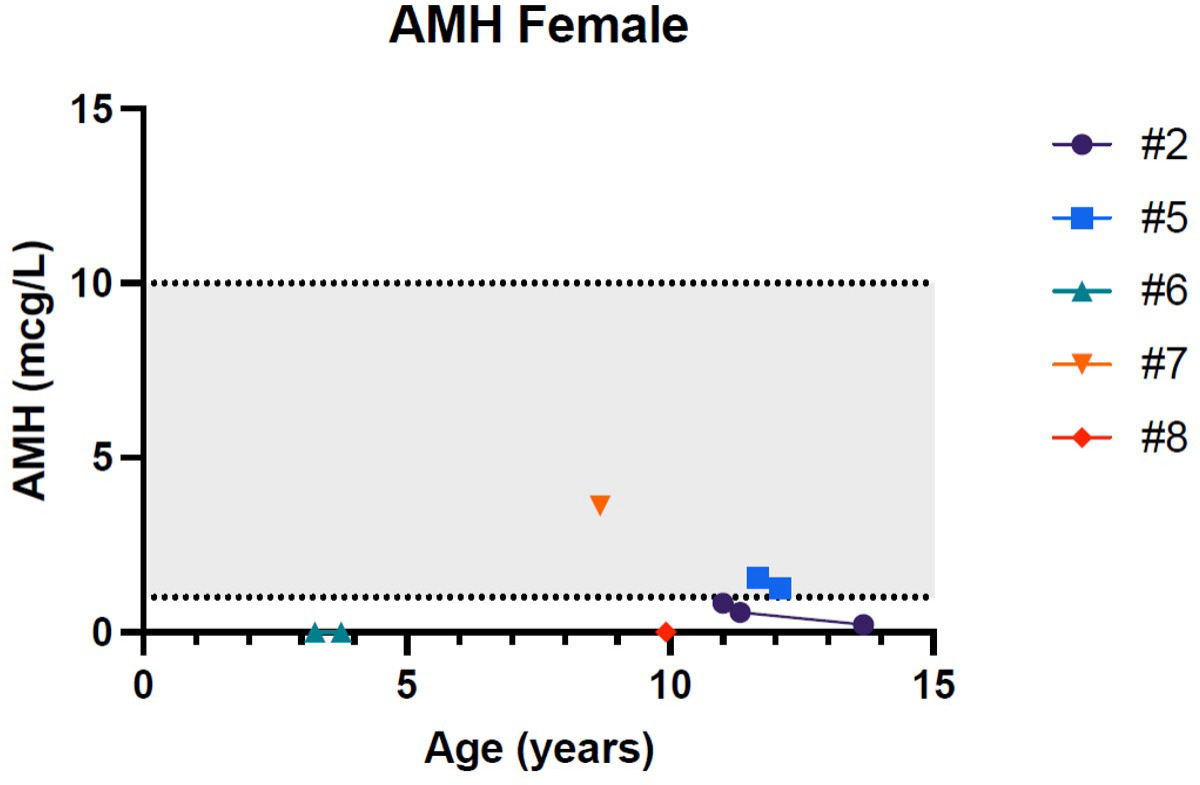
Trend of anti-Mullerian hormone in females. # stands for “patient”.

**Figure 3:**
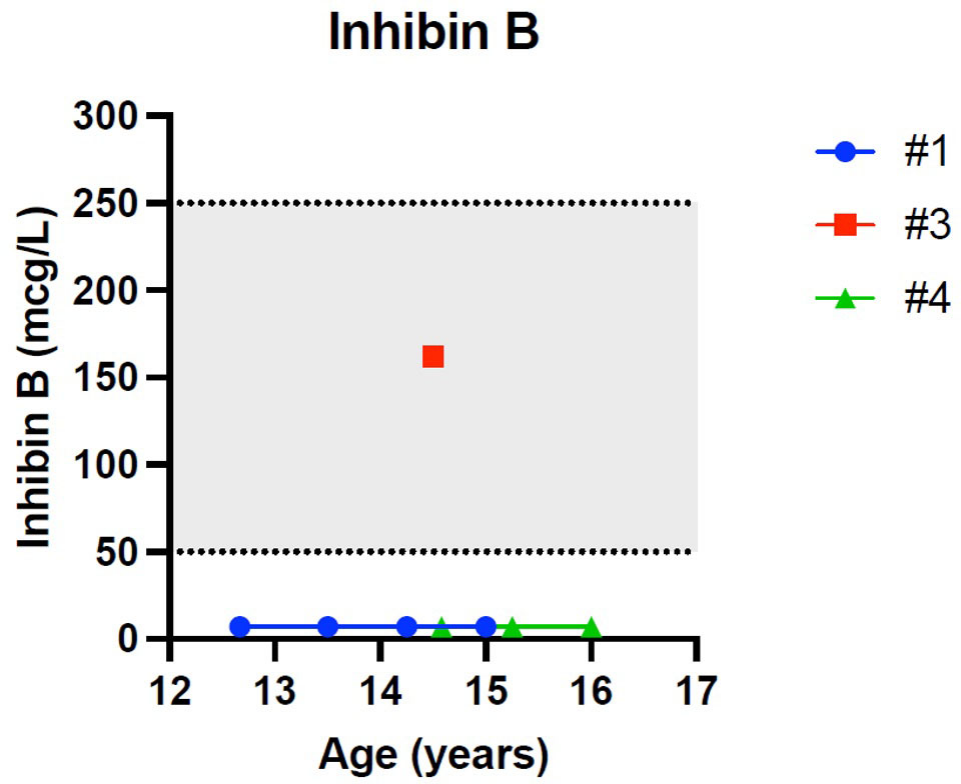
Trend of inhibin-B in males. # stands for “patient”.

**Figure 4:**
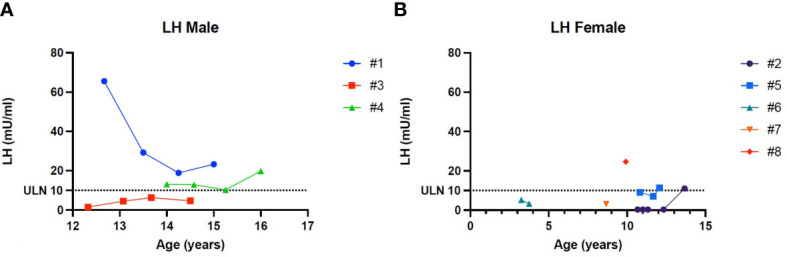
**(A)** Trend of the Luteinizing hormone in males. **(B)** Trend of the Luteinizing hormone in females. # stands for “patient”.

**Figure 5:**
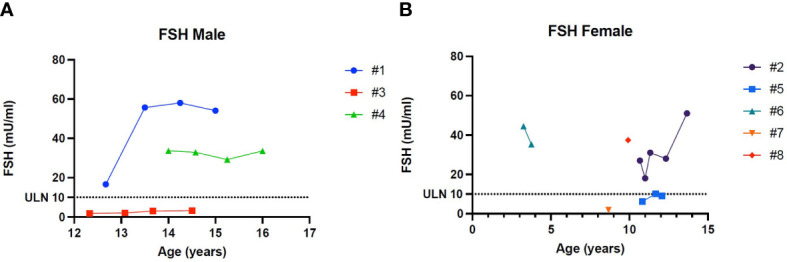
**(A)** Trend of Follicle-stimulating hormone in males. **(B)** Trend of Follicle-stimulating hormone in females. # stands for “patient”.

**Figures 1**–**5** show trends of AMH in males and females, Inhibin B, and gonadotropins respectively.

## 4 Discussion

### 4.1 Gonadal dysfunction: an underrated clinical feature

Clinical presentation in WFS1-SD is often complicated by neurological, urological, psychiatric and other endocrine dysfunctions. Some of the features are under-recognized despite their significant repercussions on prognosis. In particular, among endocrinological problems, gonadal dysfunction has been described in affected patients and referred to as a suggestive clinical feature and is therefore not included in major nor minor diagnostic criteria. Cases of hypogonadotropic hypogonadism due to hypopituitarism and hypothalamic dysfunction have been classically reported since the first patient described by Wolfram in 1938 (14, 15). Nonetheless descriptions of both primary and secondary hypogonadism are increasingly found in literature (14–18). The prevalence of hypogonadism in WFS1-SD patients varies widely from different reports. Salzano et al. analysed clinical findings in 14 patients with WFS1-SD and reported a prevalence of 7.1% for both hypogonadotropic hypogonadism and hypergonadotropic hypogonadism (16). A Spanish study on a cohort of 50 individuals observed hypogonadism in 27.8% of them (19). Simsek et al. described gonadal failure in about a third of WFS1-SD cases (20). Importantly, the low prevalence of the disease and heterogeneous clinical presentation makes it difficult to establish an accurate frequency of associated manifestations. Gonadal dysfunction may manifest subtly and variably as well. To date, gonadal failure has been observed more frequently in young male adults with heterogeneous clinical features (atrophic testes, small penis, erectile dysfunction, gynecomastia and poor secondary sexual characteristics) (2, 5, 15, 16, 19, 21). Females may often present primary amenorrhoea with low gonadotropins (secondary hypogonadism) and a review from 2018 describes normal ovarian function in female WFS1-SD patients (22). The reason for differences in gender in term of gonadal function is yet to be elucidated. Interestingly the first case of primary hypergonadotropic hypogonadism in a female patient with confirmed WFS1-SD diagnosis has been recently described in a 16 year adolescent (23). Moreover, pubertal delay is often observed in WFS1-SD adolescents alongside with growth delay due to concomitant hypopituitarism (15, 23). Nevertheless examples of successful pregnancy and delivery have been described and first reports of male fertility date back to 2013 (15, 24, 25).

### 4.2 Pathogenesis of gonadal dysfunction in WFS1-SD

Hypogonadism in Wolfram patients has been classically attributed to hypothalamic dysfunction (secondary or hypogonadotropic hypogonadism). However, recent reports have suggested that primary hypogonadism (hypergonadotropic hypogonadism) may also be part of the spectrum (14, 15, 32). Currently, the pathogenetic mechanism underlying primary gonadal failure observed in some patients remains unclear and cannot possibly be attributed to neurodegeneration or uncontrolled diabetes. However, a first hypothesis about wolframin’s role in gonadal failure has been suggested. Noormets et al. investigated the role of WFS1 gene in infertility using a murine model (33). They generated WFS1-deficient (WFS1KO) male mice and showed that reduced fertility may be related to changes in sperm morphology and reduced number of spermatogonia and Sertoli cells. On the contrary, Leydig cell number and morphology, serum testosterone and FSH concentrations did not differ between WFS1KO mice and wild-type. It is yet unclear whether wolframin underexpression impairs spermatogenesis by consequently increasing ER-stress and spermatogenic cell apoptosis. Further research is needed to clarify the mechanism through which WFS1 mutations affect gonadal cells function. Das et al. enrolled and studied clinical progression in five patients (three males, two females) with genetically confirmed WFS1-SD at a single tertiary care centre in India (17). They observed pubertal delay in 60% of patients, with primary amenorrhoea (with low gonadotropins) in both female patients (one requiring hormone therapy for pubertal initiation) and primary gonadal failure in two of the three male patients. Both of the latter showed elevated levels of gonadotropins at hormonal analysis and hormonal replacement therapy was started. Testicular biopsy revealed partly hyalinised seminiferous tubules and prominence of Leydig cells. Immunohistochemical analysis confirmed the presence of mutated wolframin, which was not significantly different from normal testis specimens on protein quantification. This may suggest gonadal cell toxicity possibly mediated by a dysfunctional protein and consequent ER-stress, as extensively described for β-pancreatic and neural cells. Although data is currently scarce, the recently proposed association between severe phenotypes and mutations causing intracellular retention, rather than loss of protein, may be suggested by reported gonadal findings (21).

These data highlight significant differences between WFS1-SD human patients and evidence from mice models with regard to laboratory and histological findings.

### 4.3 Our cohort

To our knowledge this is the first case series investigating gonadal function in paediatric patients suffering from WFS1-SD.

Despite the small cohort, consistent with the rareness of WFS1-SD, primary hypogonadism was diagnosed in 50% of patients (n=4), more specifically 67% (n=2) of males and 40% of females (n=2). These data confirm that gonadal dysfunction may be a frequent and underdiagnosed clinical feature in WFS1-SD. In particular, hypergonadotropic hypogonadism seems to be more common than previously described, involving both male and female patients. Underdiagnosis may have previously been due to short life expectancy and other more life-threatening, life-worsening, and therefore alluring clinical features.

#### 4.3.1 Gonadal dysfunction seems more frequent in males than in females

Patient 1 and Patient 4 were diagnosed with hypergonadotropic hypogonadism with a concomitant undetectable level of inhibin B, highlighting the early dysfunction of Sertoli cells, which may therefore be more affected than testosterone producing Leydig cells. In fact, patient 1 showed normal testosterone until the age of 14 years and Patient 4 had normal testosterone at the latest follow-up.

Patient 3 had a normal hormonal assessment (LH, FSH and inhibin B values) and pubertal progression. Interestingly patient 3 is the male sibling of the female Patient 5, who also presented a normal hormonal assessment including AMH and pubertal progression, suggesting a normal ovarian reserve for age and sex. This may suggest that there is a specific genotype-phenotype correlation with gonadal dysfunction but further studies on larger populations are needed to confirm this hypothesis.

Our data suggest that primary hypogonadism occurs more frequently and at a younger age in female subjects than their male counterparts. This may suggest that ovarian tissue may be more affected by wolframin dysfunction than testicular tissue.

Only one case of female primary gonadal failure in patients affected by WS has been described in literature so far, as reported by Jodoin et al. in a young adolescent patient (23). To determine if a correlation between the genotype and the type and onset of clinical manifestations in WS exists, also including sexual development issues, is complicated by the rarity of the disease.

Our experience suggests that hypergonadotropic hypogonadism in WS1 is more frequent than previously reported. Furthermore, this clinical feature seems to be even more common than hypogonadotropic hypogonadism, contrary to what has been previously described.

## 5 Conclusions

Our case series suggests that gonadal dysfunction, with potentially significant morbidity and impaired quality of life, represents a frequent and early clinical feature in WFS1-SD. The mechanisms underlying wolframin defects and gonadal failure and possible phenotype-genotype correlations have yet to be clarified. Since gonadal function appears to be a significant, not just “suggestive” feature of the disease, our proposal is that gonadal dysfunction should be included amongst clinical diagnostic criteria, as has already been proposed for urinary dysfunction (18, 34). Early diagnosis of hypogonadism may assist in an earlier clinical suspect, allowing timely diagnosis and follow-up and care of treatable associated diseases in these young patients, including hormonal replacement therapy when needed (35, 36). Periodical assessment of gonadal function and pubertal development in every patient affected by WFS1-SD is therefore also fundamental, starting from very early age. Considering that WFS-1 mutations have been described in patients with subtle clinical features and that signs and symptoms of WFS1-SD are extremely variable, the existence of patients with gonadal dysfunction as the only presenting sign cannot be ruled out. Currently, the presence of two pathological mutations in WFS1 is considered a sufficient criteria for diagnosis (according to EUROWABB guidelines) (37). Therefore, the presence of gonadal dysfunction alongside other WFS1-SD clinical features should be a significant lead towards genetic testing.

## Data availability statement

The original contributions presented in the study are included in the article/supplementary material. Further inquiries can be directed to the corresponding author.

## Ethics statement

Ethical review and approval was not required for the study on human participants in accordance with the local legislation and institutional requirements. Written informed consent to participate in this study was provided by the participants’ legal guardian/next of kin.

## Author contributions

GF, RT, and MS conceived the idea and wrote the manuscript. FA wrote the manuscript. All authors supervised the findings of this work. All authors contributed to the article and approved the submitted version.

## References

[1] Wolfram Syndrome. Orphanet. the portal for rare diseases and orphan drugs (2022). Available at: https://www.orpha.net/consor/cgi-bin/OC_Exp.php?Lng=GB&Expert=3463 (Accessed October 9, 2022).

[2] Urano F. Wolfram syndrome: diagnosis, management, and treatment. Curr Diabetes Rep (2016) 16(1):6. doi: 10.1007/s11892-015-0702-6 PMC4705145 26742931

[3] Abreu D Asada R Revilla JMP Lavagnino Z Kries K Piston DW. Wolfram syndrome 1 gene regulates pathways maintaining beta-cell health and survival. Lab Invest (2020) 100(6):849–62. doi: 10.1038/s41374-020-0408-5 PMC7286786 32060407

[4] Rigoli L Lombardo F di Bella C. Wolfram syndrome and WFS1 gene. Clin Genet (2011) 79(2):103–17. doi: 10.1111/j.1399-0004.2010.01522.x 20738327

[5] Rigoli L Caruso V Salzano G Lombardo F. Wolfram syndrome 1: from genetics to therapy. Int J Environ Res Public Health (2022) 19(6):3225. doi: 10.3390/ijerph19063225 35328914 PMC8949990

[6] Stone SI Abreu D McGill JB Urano F. Monogenic and syndromic diabetes due to endoplasmic reticulum stress. J Diabetes Complications. (2021) 35(1):107618. doi: 10.1016/j.jdiacomp.2020.107618 32518033 PMC7648725

[7] Abreu D Urano F. Current landscape of treatments for wolfram syndrome. Trends Pharmacol Sci (2019) 40(10):711–4. doi: 10.1016/j.tips.2019.07.011 PMC7547529 31420094

[8] Barrett TG Bundey SE. Wolfram (DIDMOAD) syndrome. J Med Genet (1997) 34(10):838–41. doi: 10.1136/jmg.34.10.838 PMC1051091 9350817

[9] Minton JAL Rainbow LA Ricketts C Barrett TG. Wolfram syndrome. Rev Endocr Metab Disord (2003) 4(1):53–59. doi: 10.1023/a:1021875403463 12618560

[10] Rigoli L di Bella C. Wolfram syndrome 1 and wolfram syndrome 2. Curr Opin Pediatr (2012) 24(4):512–7. doi: 10.1097/MOP.0b013e328354ccdf 22790102

[11] O’Neill MJF. OMIM®, an online catalog of human genes and genetic disorders (2011). WOLFRAM-LIKE SYNDROME, AUTOSOMAL DOMINANT; WFSL. Available at: https://omim.org/entry/614296#references (Accessed October 9, 2022).

[12] Tranebjærg L Barrett T Rendtorff N Gupta R Williams D Wright B. WFS1 wolfram syndrome spectrum disorder. In: Adam MP Mirzaa GM Pagon RA, editors. GeneReviews®. Seattle (WA: University of Washington, Seattle; 1993-2022 (2020).

[13] Gong Y Xiong L Li X Su L Xiao H. A novel mutation of WFS1 gene leading to increase ER stress and cell apoptosis is associated an autosomal dominant form of wolfram syndrome type 1. BMC Endocr Disord (2021) 21(1):76. doi: 10.1186/s12902-021-00748-z 33879153 PMC8059287

[14] Homan MR MacKay BR. Primary hypogonadism in two siblings with wolfram syndrome. Diabetes Care (1987) 10(5):664–5. doi: 10.2337/diacare.10.5.664 3677989

[15] Peden NR Gay JD Jung RT Kuwayti K. Wolfram (DIDMOAD) syndrome: a complex long-term problem in management. Q J Med (1986) 58(226):167–80. 3086928

[16] Salzano G Rigoli L Valenzise M Chimenz R Passanisi S Lombardo F. Clinical peculiarities in a cohort of patients with wolfram syndrome 1. Int J Environ Res Public Health (2022) 19(1):520. doi: 10.3390/ijerph19010520 35010780 PMC8744633

[17] Das L Rai A Mavuduru R Vaiphei K Sharma A Gupta V. Wolfram syndrome: clinical and genetic profiling of a cohort from a tertiary care centre with characterization of the primary gonadal failure. Endocrine (2020) 69(2):420–9. doi: 10.1007/s12020-020-02320-6 32350710

[18] Rigoli L Aloi C Salina A Di Bella C Salzano G Caruso R. Wolfram syndrome 1 in the Italian population: genotype–phenotype correlations. Pediatr Res (2020) 87(3):456–62. doi: 10.1038/s41390-019-0487-4 31266054

[19] Bueno GE Ruiz-Castañeda D Martínez JR Muñoz MR Alascio PC. Natural history and clinical characteristics of 50 patients with wolfram syndrome. Endocrine (2018) 61(3):440–6. doi: 10.1007/s12020-018-1608-2 29728875

[20] Simsek E Simsek T Tekgül S Hosal S Seyrantepe V Aktan G. Wolfram (DIDMOAD) syndrome: a multidisciplinary clinical study in nine Turkish patients and review of the literature. Acta Paediatr (2003) 92(1):55–61. doi: 10.1111/j.1651-2227.2003.tb00469.x 12650300

[21] de Heredia ML Clèries R Nunes V. Genotypic classification of patients with wolfram syndrome: insights into the natural history of the disease and correlation with phenotype. Genet Med (2013) 15(7):497–506. doi: 10.1038/gim.2012.180 23429432

[22] Rigoli L Bramanti P di Bella C de Luca F. Genetic and clinical aspects of wolfram syndrome 1, a severe neurodegenerative disease. Pediatr Res (2018) 83(5):921–9. doi: 10.1038/pr.2018.17 29774890

[23] Jodoin A Marchand M Beltrand J. Wolfram syndrome in a young woman with associated hypergonadotropic hypogonadism – a case report. J Pediatr Endocrinol Metab (2022) 35(12):1552–1555. doi: 10.1515/jpem-2022-0268 36100371

[24] Haghighi A Haghighi A Setoodeh A Saleh-Gohari N Astuti D Barrett TG. Identification of homozygous WFS1 mutations (p.Asp211Asn, p.Gln486*) causing severe wolfram syndrome and first report of male fertility. Eur J Hum Genet (2013) 21(3):347–51. doi: 10.1038/ejhg.2012.154 PMC3573194 22781099

[25] Kesavadev J Kumar A Shankar A Gopalakrishnan G Permutt MA Wasson J. An Asian Indian woman with wolfram syndrome on insulin pump: successful pregnancy and beyond. Diabetes Technol Ther (2011) 13(7):781–5. doi: 10.1089/dia.2010.0242 21517693

[26] Frontino G Raouf T Canarutto D Tirelli E Di Tonno R Rigamonti A. Case report: off-label liraglutide use in children with wolfram syndrome type 1: extensive characterization of four patients. Front Pediatr (2021) 9:755365. doi: 10.3389/fped.2021.755365 34970515 PMC8712700

[27] Lucas-Herald AK Mitchell RT. Testicular sertoli cell hormones in differences in sex development. Front Endocrinol (Lausanne). (2022) 13:919670. doi: 10.3389/fendo.2022.919670 35909548 PMC9329667

[28] Sowers MFR Eyvazzadeh AD McConnell D Yosef M Jannausch ML Zhang D. Anti-mullerian hormone and inhibin b in the definition of ovarian aging and the menopause transition. J Clin Endocrinol Metab (2008) 93(9):3478–83. doi: 10.1210/jc.2008-0567 PMC2567855 18593767

[29] Kanakatti Shankar R Dowlut-McElroy T Dauber A Gomez-Lobo V. Clinical utility of anti-mullerian hormone in pediatrics. J Clin Endocrinol Metab (2022) 107(2):309–23. doi: 10.1210/clinem/dgab687 PMC8764360 34537849

[30] Tal R Seifer DB. Ovarian reserve testing: a user’s guide. Am J Obstet Gynecol. (2017) 217(2):129–40. doi: 10.1016/j.ajog.2017.02.027 28235465

[31] Tanner JM. Normal growth and techniques of growth assessment. Clin Endocrinol Metab (1986) 15(3):411–45. doi: 10.1016/s0300-595x(86)80005-6 3533329

[32] Richard-Eaglin A. Male And female hypogonadism. Nurs Clinics North America. (2018) 53(3):395–405. doi: 10.1016/j.cnur.2018.04.006 30100005

[33] Noormets K Kõks S Kavak A Arend A Aunapuu M Keldrimaa A. Male Mice with deleted wolframin (Wfs1) gene have reduced fertility. Reprod Biol Endocrinology. (2009) 7(1):82. doi: 10.1186/1477-7827-7-82 19664290 PMC2734842

[34] Urano F. Wolfram syndrome iPS cells: the first human cell model of endoplasmic reticulum disease. Diabetes (2014) 63(3):844–6. doi: 10.2337/db13-1809 PMC3931391 24556864

[35] Stancampiano MR Lucas-Herald AK Russo G Rogol AD Ahmed SF. Testosterone therapy in adolescent boys: the need for a structured approach. Hormone Res paediatrics. (2019) 92(4):215–28. doi: 10.1159/000504670 31851967

[36] Nordenström A Ahmed SF van den Akker E Blair J Bonomi M Brachet C. Pubertal induction and transition to adult sex hormone replacement in patients with congenital pituitary or gonadal reproductive hormone deficiency: an endo-ERN clinical practice guideline. Eur J Endocrinol (2022) 186(6):G9–G49. doi: 10.1530/EJE-22-0073 35353710 PMC9066594

[37] Wolfram Syndrome Guideline Development Group. Management of wolfram syndrome. a clinical guideline (2014). Available at: http://www.orpha.net/national/data/IE-EN/www/uploads/Wolfram2014.pdf (Accessed October 9, 2022).

